# The products from fermentation of wheat bran fiber by *Auricularia polytricha* strain and the effects of the products on rheological properties of dough sheet

**DOI:** 10.1002/fsn3.1366

**Published:** 2020-02-06

**Authors:** Shiyu Jiang, Li Li, Limin Li, Xueling Zheng, Zhengzhe Li, Xin Song

**Affiliations:** ^1^ College of Grain and Food Science Henan University of Technology Zhengzhou China

**Keywords:** *Auricularia polytricha*, degradation products, intermediate products, metabolites, rheological properties, wheat bran fiber

## Abstract

Being classified within the *Basidiomycota*, *Auricularia polytricha* has been proved to degrade lignocellulose, a major component of wheat bran fiber. During the fermentation of lignocellulose by *A. polytricha* strain, a large number of intermediate products are produced, which affect the further degradation of lignocellulose. Therefore, it is essential to analyze the fermentation intermediates for study on the degradation mechanism of wheat bran fiber. In this study, the effectiveness of fermentation of wheat bran fiber by *A. polytricha* strain was confirmed via scanning electron microscopy. Additionally, the results of gas chromatography–mass spectrometry indicated that the *A. polytricha* strain could degrade wheat bran fiber and produce several aromatic compounds, and that the number of products obtained after 7 days of fermentation was significantly lower than that after 3 days of fermentation. It has also been demonstrated that diisooctyl phthalate and 9‐octadecenamide belong to metabolites produced during the fermentation of wheat bran fiber, by culturing *A. polytricha* with wheat bran fiber and glucose as carbon source, respectively. Moreover, by conducting an ultraviolet wavelength scanning of the culture liquid containing vanillin fermented by *A. polytricha*, it has been indicated that the strain could degrade vanillin and further demonstrated that the strain has the ability to degrade wheat bran fiber. Furthermore, adding the products of wheat bran fiber fermented for 3 days by *A. polytricha* could improve the elasticity of the dough sheet.

## Graphical abstract

This study proved that *Auricularia polytricha* 5.584 has the ability to degrade wheat bran fiber. The metabolites of *A. polytricha* 5.584 fermenting wheat bran fiber were determined by culturing the strain in different media. This study lays a foundation for study on the degradation mechanism of wheat bran fiber by *A. polytricha* 5.584.

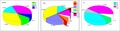

## 1 INTRODUCTION

Wheat bran fiber belongs to lignocellulose, which comprises of three major components: cellulose, hemicellulose, and lignin. Lignocellulose is one of the most abundant materials in nature and represents an extremely large quantity of renewable bioresources available on the planet, with numerous applications (Kumar & Sharma, 2017). In the structure of lignocellulose, the carbohydrate polymers, cellulose, and hemicellulose are constrained to the lignin by hydrogen and covalent bonds (Vorwerk et al., 2004; Imen et al., 2009). Lignin consists of a largely amorphous phenylpropanoid polymer that acts similarly to glue by filling the space around the cellulose and hemicellulose structures, binding them together (Vorwerk et al., 2004; Hamelinck et al., 2005; Perilä, 2010). Therefore, recalcitrant and complex structures in wheat bran fiber are obstacles for the release of dietary fiber, bioactive compounds, and other nutrients.

According to the mechanism of degrading biomass, fungi that are active in the biodegradation of wood can be classified into three main groups: white‐rot, brown‐rot, and soft‐rot (Isroi et al., 2011). Their names are derived from the specific bleaching process that occurs during the fungi degradation of wood (ten Have & Teunissen, 2001). The fungus selected in our experiment is *Auricularia polytricha* strain (CGMCC 5.584), which belongs to white‐rot fungi. The lignin‐decomposing enzymes (lignin peroxidase, manganese peroxidase, and laccase) produced by white‐rot fungi can effectively convert cellulose, hemicellulose, and lignin into CO_2_ and H_2_O (Ander, 2010). Furthermore, they are the most effective and dominant lignin‐degrading microorganisms identified so far.

Compared with physical, chemical, and combined processes, biological fermentation has potential advantages, such as lower pollution generation, lower energy requirement, and reduced inhibitors production under mild reaction conditions (Manickam et al., 2018). The microorganisms predominantly responsible for lignocellulose biodegradation are species of white‐, brown‐, and soft‐rot filamentous fungi (Rhizopus, Trichoderma, Aspergillus, Penicillium, Fusarium, Phanerocheate, etc.), bacteria (Bacillus, Clostridium, Cellulomonas, Fibribacter, Pseudomonas, Thermoanaerobaterium, etc.), and actinomycetes (Thermoactinomyces, Thermomonospora, etc.) (Farkas et al., 2019). During the fermentation of lignocellulose by microorganism, a large number of intermediate products are produced, which affect the further degradation of lignocellulose. Therefore, it is essential to analyze the fermentation intermediates for study on the degradation mechanism of wheat bran fiber. Based on the characteristics of high resolution and high sensitivity of GC‐MS, the intermediate products of wheat bran fiber fermented by *A. polytricha* 5.584 were detected by GC‐MS. Additionally, based on the advantages of scanning electron microscopy (SEM), such as high resolution, wide and adjustable magnification range, large depth of field, and simple sample preparation, the differences between unprocessed wheat bran, enzymatically hydrolyzed wheat bran fiber, and fermented wheat bran fiber were observed by SEM. Moreover, the ability of *A. polytricha* 5.584 to utilize the aromatic compounds was verified by adding an intermediate product into liquid degradation medium and then detecting the ultraviolet spectra of the fungal solution at different times. Furthermore, the product of wheat bran fiber fermented by *A. polytricha* 5.584 was added into flour, and the change of elasticity and viscosity of dough sheet was studied by HAAKE rheometer. This study laid a foundation for studying the metabolic pathway of wheat bran fiber degradation by *A. polytricha* 5.584 and the mechanism of lignocellulose degradation.

## 2 MATERIALS AND METHODS

### 2.1 Chemicals and materials

N,O‐Bis(trimethylsilyl) trifluoro‐acetamide with trimethyl chlorosilane (BSTFA) (99%, contains 1% TMCS) and vanillin were purchased from Sigma‐Aldrich (Taufkirchen, Germany); ethyl acetate (≥99.5% purity) and NaOH were supplied by Tianjin Tianli Chemical Reagent Co., Ltd.; potato dextrose agar medium and yeast extract were provided by Beijing Aoboxing Biotechnology Co., Ltd.; α‐amylase and alkaline protease were purchased from Beijing Solarbio Science and Technology Co., Ltd.; HCl and (NH_4_)_2_SO_4_ were supplied by Luoyang Haohua Chemical Reagent Co., Ltd.; VB_1_ was provided by Huazhong Pharmaceutical Co., Ltd.; oxytetracycline was purchased from Nanjing Extreme Aquarium Products Co., Ltd.; standards of Pyridine (≥99.5% purity), 1,4‐dioxane (≥99.5% purity), D‐glucose, KH_2_PO_4_, K_2_HPO_4_, FeSO_4_, MgSO_4_, MnSO_4,_ and CaCl_2_·2H_2_O were supplied by Tianjin Kermel Chemical Reagent Co., Ltd.; wheat bran was provided by Henan Zhonghe Pure Powder Co., Ltd.; the flour of Shenxiang Teyi was purchased from Zhengzhou Haijia Food Co., Ltd.

### 2.2 Microorganism

We cultured *A. polytricha* 5.584, 5.583, 14,121, 50,017, 5.246, 5.247, 336,040, 336,039, and 143,171. We found that *A. polytricha* 5.584 grew vigorously and had superior activity. Moreover, *A. polytricha* is an edible jelly fungus. Therefore, *A. polytricha* 5.584 was selected to ferment wheat bran fiber. *A. polytricha* 5.584 was purchased from the Institute of Microbiology, Chinese Academy of Sciences.

### 2.3 Medium and growth condition

Slant medium (g/100 ml): PDA 3.7, KH_2_PO4 0.25, MgSO_4_ 0.15, VB_1_ 0.05, oxytetracycline 0.02;

Liquid medium (g/100 ml): glucose 2.0, yeast extract 0.20, KH_2_PO_4_ 0.25, MgSO_4_ 0.15, VB_1_ 0.05, oxytetracycline 0.02;

Liquid degradation medium (g/100 ml): wheat bran 5, K_2_HPO_4_ 0.1, KH_2_PO_4_ 0.1, MgSO_4_ 0.02, FeSO_4_ 0.005, (NH_4_)_2_SO_4_ 0.2, MnSO_4_ 0.002, CaCl_2_·2H_2_O 0.01;

Glucose medium (g/100 ml): glucose 2.0, K_2_HPO_4_ 0.1, KH_2_PO_4_ 0.1, MgSO_4_ 0.02, FeSO_4_ 0.005, (NH_4_)_2_SO_4_ 0.2, MnSO_4_ 0.002, CaCl_2_·2H_2_O 0.01.

A three‐stage cultivation technique was applied in this experiment. First, *A. polytricha* 5.584 was inoculated on the slant medium and cultured for 5 days at 27°C. Second, the single colonies were inoculated into the liquid medium and cultured for 6 days at 27°C and 180 rpm. Third, 2 ml of the fungal solution was taken and centrifuged at 4,000 rpm for 10 min. After discarding the supernatant, the cells were washed three times with sterile water, resuspended in 2 ml of sterile water, and inoculated into 100 ml of the liquid degradation medium and glucose medium (control) for 7 days under culture conditions of 27°C and 180 rpm, respectively.

### 2.4 Preparation of wheat bran fiber

Wheat bran was first pulverized with a multifunction crusher, passed through a 40‐mesh screen, mixed with distilled water in a ratio of 1:10, and wet‐ground for 25 min using a colloid mill. After drying, the wheat bran was put into a beaker containing distilled water (the ratio was 1:10) and was heated at 95°C for 30 min. After the wheat bran was cooled to about 50°C, its pH was adjusted using HCl to 5.6, and the wheat bran was heated to about 100°C. Subsequently, α‐amylase (1.5%) was added and mixed for 30 min. After the wheat bran was cooled to about 50°C, its pH was adjusted to 5.6 with NaOH and mixed the wheat bran thoroughly. Alkaline protease (3%) was then added, and the mixture was stirred for 2 hr and heated to 100°C to inactivate the enzyme. The residue was finally washed with distilled water and dried for 10 hr at 60°C in a drying oven to produce the wheat bran fiber.

### 2.5 Preparation of fermented wheat bran fiber

Take the culture solution which has been fermented for 3 and 7 days, centrifuged at 12,000 rpm for 10 min. After the supernatant was discarded, wash the wheat bran fiber with water and dried for 10 hr at 60°C in a drying oven.

### 2.6 Quantitative analysis of wheat bran

The contents of starch, protein, ash, and fiber in wheat bran, enzymatic hydrolyzed wheat bran fiber, and fermented wheat bran fiber were determined. The determination of fiber refers to the national standard of the people's Republic of China GB 5009.88–2014; the determination of starch refers to GB/T 20378–2006; the determination of protein refers to GB 5009.5–2016; the determination of ash refers to GB 5009.4–2016.

### 2.7 Scanning electron microscopy

The wheat bran, enzymatically hydrolyzed wheat bran fiber, and fermented wheat bran fiber were fixed on copper carriers with double coated tape and coated with gold before they were observed with SEM at 3.00 kv.

### 2.8 Extraction of the fermentation products of wheat bran fiber

The fungal solution was first taken and centrifuged at 12,000 rpm for 10 min. After the suspension was discarded by freezing–thawing, the pH was adjusted to 2.0 by mixing bacterial solution thoroughly with HCl. The supernatant was then extracted with twice the volume of ethyl acetate. After mixing vigorously, the organic layer was separated and concentrated into about 1 ml in a rotary evaporator under the condition of 35°C and 100 rpm. After the concentrate was dried with N_2_, add 100 μL of 1,4‐dioxane, 10 μL of pyridine, and 50 μL of BSTFA and heat at 60°C for 30 min in a thermostat‐controlled water bath. After the concentrate was cooled to room temperature, dry with N_2_ and add 1 ml ethyl acetate. It was then centrifuged at 1,000 rpm for 3 min for GC‐MS analysis.

### 2.9 Chromatographic conditions

The GC‐MS analysis was carried out with an Agilent Technologies 7890A. The column used was Hp‐5ms (30 m × 0.32 mm, 0.50 μm). The initial column temperature was 50°C. After 5 min, the temperature was increased to 290°C at a rate of 8°C/min and kept constant for 8 min. Helium was used as carrier gas at a flow rate of 1 ml/min. The injection volume was 1 μL, the split ratio was 20:1, and the solvent delay time was set to 5 min. Intermediates were identified based on their mass spectra (the detector is Agilent technologies 5975C) and NIST 11.L library data.

### 2.10 Degradation of vanillin by A. polytricha 5.584

Vanillin with the concentration of 0.1 g/L was added to the liquid medium. After inoculation, 100 μL of culture medium was added to 5 ml of sterile water as sample every day. The sample was observed with ultraviolet spectral scanning in the wavelength range of 600–190 nm using an ultraviolet spectrophotometer. The ability of degradation of vanillin by *A. polytricha* 5.584 was verified by observing the change of characteristic absorption peak.

### 2.11 The preparation of dough sheet

The method of making fresh dough sheet refers to the industry standard SB/T 10137–1993. Weigh 100 g flour, adjust the water content of flour to 35% with distilled water. The dough was formed using a dough mixer (Modle JHMZ; Beijing, China), and the mixing time was 7 min. The prepared dough was allowed to rest in a clean basin covered with wet gauze for 15 min to let the flour and starches hydrate. The dough crumbles were then sheeted on a machine (Model JMTD; Beijing, China) at 2 mm to smooth and firm it for five times and fed another five times between the rollers. Decrease the gap between rollers each time until the thickness was 1 mm. The dough sheet was pressed into 4 cm in diameter and covered with plastic film.

### 2.12 Rheological measurement of dough sheet

In the linear viscoelastic region, frequency scanning experiments were carried out by using Haake mixer to determine the variation of elastic modulus, viscous modulus, and loss factor with frequency. A P35TiL rotor was used. The distance between two plates was 1 mm. The diameter of plate was 35 mm. The temperature was 25°C. The fixed strain was 0.5%. The frequency range was 0.1–10 Hz.

## 3 RESULTS AND DISCUSSION

### 3.1 Quantitative analysis of wheat bran

The contents of starch, protein, ash, and fiber in wheat bran, enzymatic hydrolyzed wheat bran fiber, and fermented wheat bran fiber were determined. The result is as follows: the component composition of wheat bran is starch 16.86%, protein 13.5%, ash 4.5%, and fiber 52.83%; the component composition of enzymatic hydrolyzed wheat bran fiber is starch 9.64%, protein 6.01%, ash 1.9%, and fiber 81.12%; the component composition of fermented wheat bran fiber for 3d is starch 3.86%, protein 5.77%, ash 1.5%, and fiber 86.85%; the component composition of fermented wheat bran fiber for 7d is starch 2.98%, protein 5.53%, ash 1.4%, and fiber 87.17%.

### 3.2 Analysis of SEM

**Figure 1:**
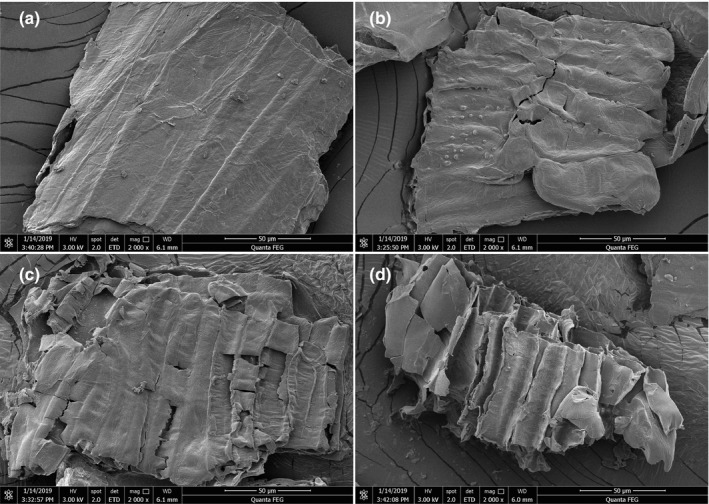
The scanning electron microscopy of the unprocessed wheat bran, enzymatically hydrolyzed wheat bran fiber and fermented wheat bran fiber by *Auricularia polytricha* 5.584 for 3 d and 7 d (a. unprocessed wheat bran (2000×); b. enzymatically hydrolyzed wheat bran fiber (2000×); c. fermented wheat bran fiber by *A. polytricha* 5.584 for 3 d (2000×); d. fermented wheat bran fiber by *A. polytricha* 5.584 for 7 d (2000×))

SEM is a new type of electronic optical instrument which has been applied extensively in biology, medicine, and metallurgy. Due to its unparalleled superiority compared to traditional microscope, which includes high resolution, wide and adjustable magnification range, large depth of field, and simple sample preparation, it has promoted the development of these related fields. In this study, the unprocessed wheat bran, enzymatically hydrolyzed wheat bran fiber, and fermented wheat bran fiber by *A. polytricha* 5.584 were observed via SEM, respectively. The SEM images are presented in Figure 1.

SEM images demonstrated that unprocessed wheat bran had complete shape, compact texture, smooth appearance, and nonporous structure inside (Figure 1a). After enzymatic hydrolysis, the surface of the wheat bran fiber was slightly loosened, with slight “corrosion” marks. The outer layer was partially fractured and slightly inhomogeneous pore structure appeared inside (Figure 1b). The surface of the wheat bran fiber which had been fermented by *A. polytricha* 5.584 for 3 days was more porous, with more obvious sign of corrosion. A residual prismatic skeleton structure also appeared (Figure 1c). After fermenting for 7 days by *A. polytricha* 5.584, a three‐dimensional fluffy state emerged in the wheat bran fiber. The surface ulceration, inner pore diameter increased, and the specific surface area increased significantly (Figure 1d).

The results of SEM indicated that enzymatic hydrolysis and fermentation could effectively modify the internal structure of wheat bran. There was a correlation between the effectiveness of the enzymatic hydrolysis and decrease in starch and protein content in the wheat bran after addition of α‐amylase and alkaline protease. The effectiveness of fermentation might relate to the degradation of insoluble dietary fiber caused by the conversion of its carbon and nitrogen. The macromolecular components of dietary fiber are decomposed into soluble small molecular compounds, which can enhance the binding capacity with water, improve the water holding capacity, oil holding capacity and swelling capacity, thus improving the physicochemical properties of wheat bran fiber. It has been found that the attack process of bacteria on wood could be divided into three stages: eroding, trenching, and drilling. Bacteria grow into the thin layer in the middle of wood fiber first, causing erosion of the fiber wall, and then trench in the fiber wall (Daniel et al., 1987). In the initial stage, alkalophilic bacteria act on wheat straw lignocellulose by invading the inside of wheat straw tissue, reproducing and aggregating in parenchyma cells and fiber cell lumen. The parenchymal cells are decomposed or even completely destroyed, and then, the intercellular layer of the most concentrated part of lignin in the fibrous cell wall is gradually decomposed (Zhang et al., 2002). These degradation pathway and mechanism of lignocellulose by bacteria have certain reference values to study on the degradation of wheat bran fiber by *A. polytricha*.

### 3.3 Analysis of intermediates of fermenting wheat bran fiber

GC‐MS is a method that combines the advantages of gas chromatography and mass spectrometry, including high resolution and sensitivity, respectively. Being widely used in the research of the separation and identification of complex components, it is an effective instrument for both the qualitative and quantitative determination of drugs and metabolites in biological samples. It has been proven as an appropriate technology for the analysis of low molecular weight compounds released during lignocellulose degradation (Ksibi et al., 2003; Hernández‐Coronado et al., 2015). For instance, Raj, A. identified the metabolites by GC‐MS from kraft lignin degradation by three *Bacillus* sp. (Raj et al., 2007). However, it is difficult to detect oligomers and trimers by GC‐MS due to their high‐boiling points (Takada et al., 2004).

**Figure 2:**
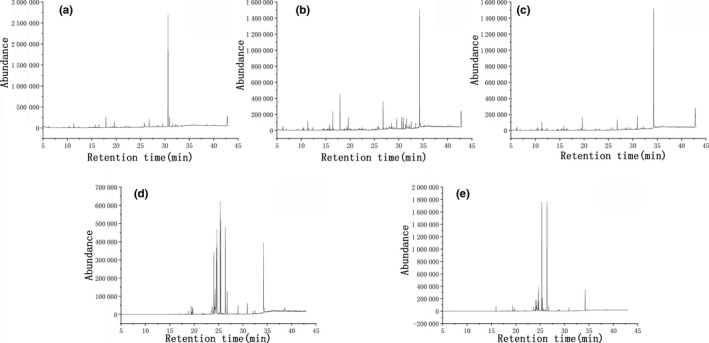
The TIC of compounds from *Auricularia polytricha* 5.584 cultured in liquid degradation medium and glucose medium (a. the TIC of wheat bran fiber fermented by *A. polytricha* 5.584 for 0 d in liquid degradation medium; b. the TIC of wheat bran fiber fermented by *A. polytricha* 5.584 for 3 d in liquid degradation medium; c. the TIC of wheat bran fiber fermented by *A. polytricha* 5.584 for 7 d in liquid degradation medium; d. the TIC of wheat bran fiber fermented by *A. polytricha* 5.584 for 3 d in glucose medium; e. the TIC of wheat bran fiber fermented by *A. polytricha* 5.584 for 7 d in glucose medium)

The total ion chromatogram (TIC) of the control group (without inoculation) and the experimental group (wheat bran fiber fermented for 3 and 7 days by *A. polytricha* 5.584) are displayed in Figure 2a–c. The retention time (RT) and the observed chemical compounds of the two group are depicted in Table 1. With regard to the control group, 5 aromatic compounds were detected (Figure 2a), including 2‐hydroxy‐5‐methylacetophenone (RT 16.45 min), vanillin (RT 17.90 min), 2,4‐di‐tert‐butylphenol (RT 19.66 min), 2,2′‐methylenebis(6‐tert)‐butyl‐4‐methylphenol (RT 30.92 min), and dioctyl phthalate (RT 32.14 min). Except for 2,2′‐methylenebis(6‐tert)‐butyl‐4‐methylphenol, the rest were all single aromatic compounds, which could be attributed to chemical oxidation of wheat bran fiber due to agitation and slight aeration in the control group. Many acid‐type compounds and ester were also identified in the control group. Figure 2b was the TIC of wheat bran fiber fermented by *A. polytricha* 5.584 for 3 days. Compared with the control group, the number of peaks increased significantly, including 1,3‐xylene (RT 6.24 min), 1,2‐xylene (RT 6.95 min), 3,5‐dimethylbenzaldehyde (RT 14.65 min), 2‐methoxy‐4‐vinylphenol (RT 16.45 min), 3,5‐dimethoxy‐4‐hydroxybenzaldehyde (RT 21.88min), 1,2‐benzenedicarboxylic acid bis (2‐methylpropyl) ester (RT 24.66 min), and diisooctyl phthalate (RT 32.14 min). Besides low molecular weight aromatic compounds, some acid‐type compounds, esters, and amides were depicted. The results indicated that many low molecular compounds were produced after the fermentation of wheat bran fiber by *A. polytricha* 5.584. The peak at RT 24.66 min was identified as 1,2‐benzenedicarboxylic acid bis (2‐methylpropyl) ester, which was also detected in studies on the depolymerisation of lignosulfonate by peroxidase of the white‐rot *basidiomycete* (Shin & Lee, 1999)and in the photodegradation of lignin from black liquor (Ksibi et al.). The compounds detected in the extracts of the fermentation products by *A. polytricha* 5.584 for 7 days were significantly fewer than those for 3 days (Figure 2c). Both vanillin and 3,5‐dimethoxy‐4‐hydroxybenzaldehyde were found in the fermentation products of wheat bran fiber by *A. polytricha* 5.584 for 3 days while only vanillin was detected in the control group. However, vanillin and the 3,5‐dimethoxy‐4‐hydroxybenzaldehyde were not detected in the fermentation products of wheat bran fiber by *A. polytricha* 5.584 for 7 days, which indicated that the strain could degrade vanillin and 3,5‐dimethoxy‐4‐hydroxybenzaldehyde.

**Table 1:** Intermediates of wheat bran fiber fermented by *Auricularia polytricha* 5.584 in the liquid degradation medium (A. 0 day; B. 3 days; and C. 7 days)

No.	RT	Compounds	A (0 d)	B (3 d)	C (7 d)
A1	6.24	1,3‐xylene	−	+	−
A2	6.95	1,2‐xylene	−	+	−
A3	14.65	3,5‐dimethylbenzaldehyde	−	+	−
A4	16.45	2‐methoxy‐4‐vinylphenol	−	+	−
A5	16.45	4‐hydroxy‐3‐methylacetophenone	−	−	+
A6	16.45	2‐hydroxy‐5‐methylacetophenone	+	−	−
A7	17.90	Vanillin	+	+	−
A8	19.66	2,4‐di‐tert‐butylphenol	+	+	+
A9	21.88	3,5‐dimethoxy‐4‐hydroxybenzaldehyde	−	+	−
A10	24.66	1,2‐benzenedicarboxylic acid bis(2‐methylpropyl)ester	−	+	−
A11	25.74	*N*‐hexadecanoic acid	+	+	−
A12	25.83	1,2‐benzenedicarboxylic acid, butyl 2‐ethylhexyl ester	−	−	+
A13	26.79	Hexadecanoic acid, trimethylsilyl ester	+	+	+
A14	28.09	Octadecanoic acid	+	+	−
A15	29.57	Methoxyacetic acid,2‐tetradecyl ester	+	+	−
A16	30.64	Hexanedioic acid,bis(2‐ethylhexyl)ester	+	−	−
A17	30.92	2,2′‐methylenebis(6‐tert)‐butyl‐4‐methylphenol	+	+	+
A18	32.14	diisooctyl phthalate	−	+	+
A19	32.14	dioctyl phthalate	+	−	−
A20	34.25	9‐octadecenamide,(z)‐	−	−	+
A21	34.25	13‐docosenamide,(z)‐	−	+	−

Out of the 14 kinds of aromatic compounds detected by GC‐MS, a total of 5 kinds were in the control group, and the rest were produced during the fermentation of wheat bran fiber by *A. polytricha* 5.584, including A1, A2, A3, A4, A5, A9, A10, A12, and A18. Additionally, the number of peaks appeared on TIC representing the products of wheat bran fiber fermented by *A. polytricha* 5.584 for 7 days was smaller than those for 3 days, which indicated that several intermediates could be further degraded.

### 3.4 Analysis of metabolites of wheat bran fiber

To further explore the metabolites of the fermentation of wheat bran fiber by *A. polytricha* 5.584, replace the wheat bran fiber in the liquid degradation medium with 2 g glucose. The concentration of glucose was 2g / 100mL. The fermentation metabolites of wheat bran fiber by *A. polytricha* 5.584 were determined by comparing them with the intermediates.

The TIC of the wheat bran fiber fermented by *A. polytricha* 5.584 in glucose medium for 3 and 7 days is displayed in Figure 2d–e, and the identification results of the main peaks are shown in Table 2. Among the peaks, 4 kinds of substances detected were also the intermediates of the wheat bran fiber fermented by *A. polytricha* 5.584 in the liquid degradation medium, including hexadecanoic acid, trimethylsilyl ester (RT 26.78 min), 2,2′‐methylenebis (6‐tert) ‐butyl‐4‐methylphenol (RT 30.91 min), diisooctyl phthalate (RT 32.13 min), and 9‐octadecenamide (RT 34.23 min). However, hexadecanoic acid, trimethylsilyl ester, and 2,2′‐methylenebis(6‐tert)‐butyl‐4‐methyl phenol were also detected in the control group, which indicated that both the substances were not metabolites of wheat bran fiber fermented by *A. polytricha* 5.584. Hence, diisooctyl phthalate and 9‐octadecenamide were metabolites of wheat bran fiber fermented by *A. polytricha* 5.584. However, whether diisooctyl phthalate and 9‐octadecenamide were degradation products of that process requires further studies.

**Table 2:** Products from fermentation by *Auricularia polytricha* 5.584 cultured in glucose medium (D. 3 days and E. 7 days)

No.	RT^a^	Compounds	D (3 d)	E (7 d)
C1	26.78	Hexadecanoic acid, trimethylsilyl ester	+	+
C2	29.00	Octadecanoic acid, trimethylsilyl ester	+	+
C3	30.91	2,2′‐methylenebis(6‐tert)‐butyl‐4‐methylphenol	+	+
C4	32.13	diisooctyl phthalate	+	−
C5	34.23	9‐octadecenamide,(z)‐	+	+

### 3.5 Analysis of aromatic compounds in intermediates of wheat bran fiber

**Figure 3:**
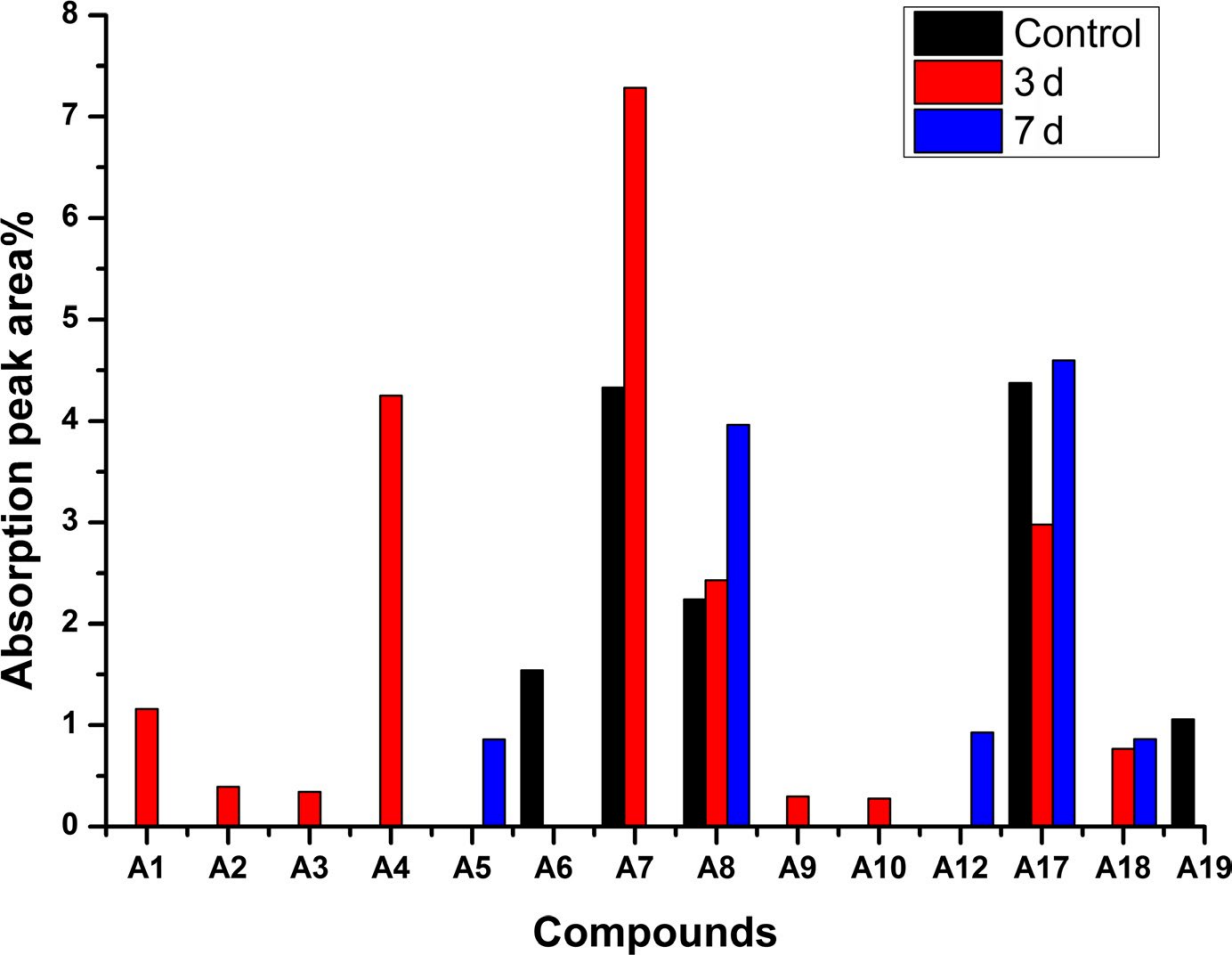
The changes in peak area of 14 aromatic compounds detected in the process of fermentation of wheat bran fiber by *Auricularia polytricha* 5.584

**Figure 4:**
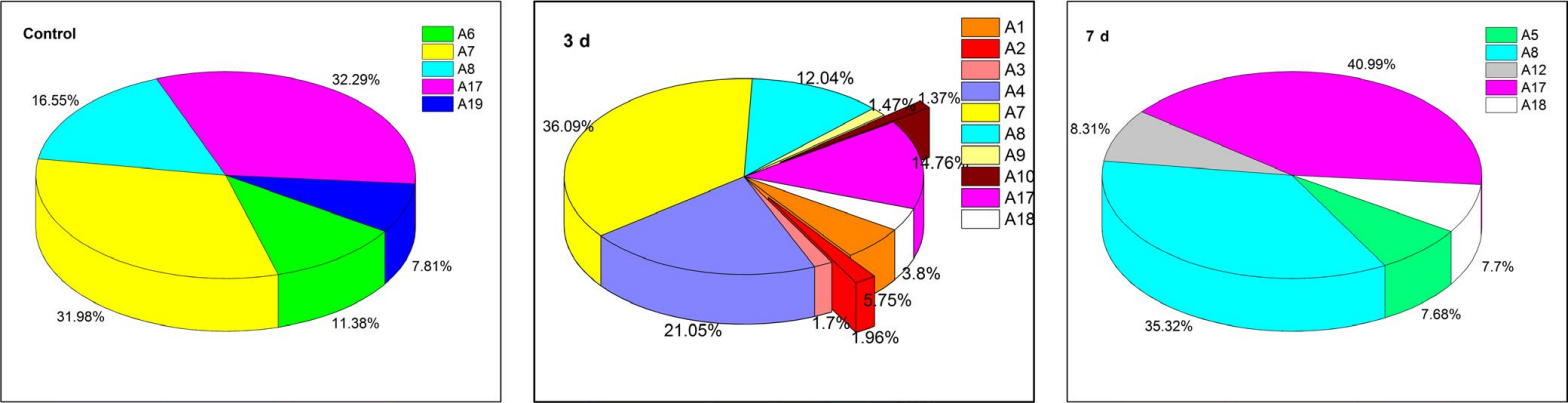
The proportion of the peak area of aromatic products during fermenting wheat bran fiber by *Auricularia polytricha* 5.584

The peak area changes of 14 aromatic compounds (A1, A2, A3, A4, A5, A6, A7, A8, A9, A10, A12, A17, A18, A19) detected in the control group and the experimental group, as well as the proportion of each substance, are exhibited in Figures 3 and 4, respectively. The corresponding label of each substance is the same as it is in Table 1.

A large amount of monocyclic aromatic compounds accumulated in the products of the fermentation of wheat bran fiber for 3 days (Figure 3), including mainly A1, A4, A7, A8, and A18. Among these compounds, vanillin was the most abundant. Interestingly, vanillin was also detected in the control group, but not detected in the fermentation products of wheat bran fiber by *A. polytricha* 5.584 for 7 days, which indicated that vanillin was completely decomposed before 7 days. Similarly, A3, A4, A9, and A10 appeared in the fermentation products for 3 days and disappeared in the fermentation products for 7 days, which showed that these substances could be absorbed and utilized in the process of fermentation of wheat bran fiber.

Figure 4 shows the proportion of each aromatic compound of the control group and the experimental group. In terms of the control group, three of the aromatic compounds with the highest amount were A17 (32.29%), A7 (31.98%), and A8 (16.55%). The content of each compound changed significantly after the fermentation of wheat bran fiber for 3 days, the major compounds were A7 (36.09%), followed by A4 (21.05%), A17 (14.76%), and A8 (12.04%). After the fermentation of wheat bran fiber for 7 days, the major compounds were A17 (40.99%) and A8 (35.32%), and the peak areas of the two substances accounted for 76.31% of the total aromatic compound peak area. Additionally, A8 and A17 were detected in both the control group and the experimental group, and the peak area first decreased and then increased, which indicated that wheat bran fiber not only could be utilized but also could produce these intermediates in the process of fermentation. Presumably, vanillin could be produced by the cleavage of the guaiacyl (G), and the 3,5‐dimethoxy‐4‐hydroxybenzaldehyde could be produced by the cleavage of the syringyl structure. Furthermore, it was possible that other aromatic compounds could be produced during the fermentation of wheat bran fiber. However, these substances could not be identified due to limited measurement range of GC‐MS. Therefore, in the following studies, liquid chromatography–mass spectrometry (LC‐MS) and nuclear magnetic resonance (NMR) could be considered to identify and analyze the fermentation products of wheat bran fiber.

### 3.6 Analysis of ultraviolet absorption spectrum

Ultraviolet absorption spectra are produced by molecules or ions absorbing ultraviolet light, which can be used to analyze the composition, content, and structure of compounds. Ultraviolet‐visible spectroscopy has been used to study the changes of chromophores during pulping and bleaching, as well as to determine the chromophores in lignin (Dence, 1992). The intermediate products that affect the degradation of lignocellulose include vanillin, 3,5‐dimethoxy‐4‐hydroxybenzaldehyde, p‐hydroxybenzoic acid, catechol, and protocatechuic acid. Vanillin was selected as a single carbon source to culture *A. polytricha* 5.584, and observing the changes in maximum absorption peak via the detection of the ultraviolet spectra for fermentation liquid at different times. This degradation characteristics could further prove the ability of *A. polytricha* 5.584 degrade vanillin, so as to better understand the mechanism of degradation of wheat bran fiber by *A. polytricha* 5.584.

**Figure 5:**
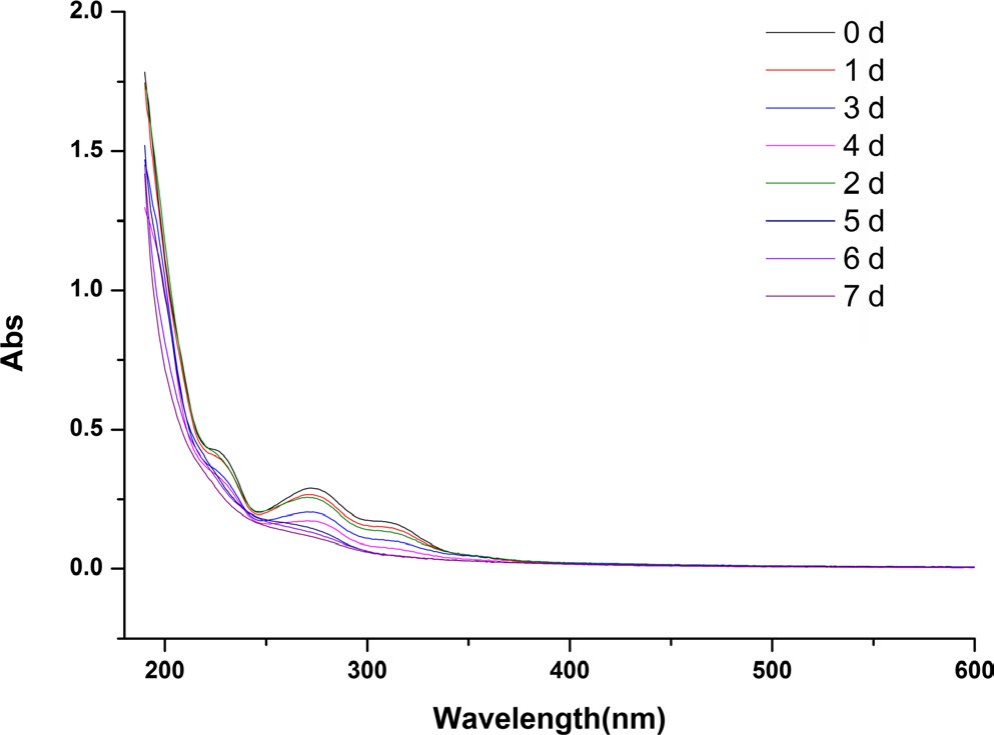
The ultraviolet spectrogram of fermentation liquid containing vanillin degraded by *Auricularia polytricha* 5.584 for different culture times

In the analysis of GC‐MS, vanillin disappeared in the fermentation products of wheat bran fiber by *A. polytricha* 5.584 for 7 days. The ultraviolet spectrogram of fermentation liquid containing vanillin degraded for different culture times is displayed in Figure 5. It can be seen from the figure that there were absorption peaks of untreated vanillin (0 days) near 247, 272, and 305 nm. However, with the prolongation of culture time, the maximum absorption peak of vanillin decreased gradually in terms of peak height. This phenomenon indicated that the concentration of vanillin in the medium decreased gradually. Additionally, the absorption peak at 247 and 272 nm disappeared after 5 days of degradation, and that at 305 nm disappeared after 6 days of degradation, indicating that vanillin was completely degraded by *A. polytricha* 5.584 within 6 days. Interestingly, the results of the ultraviolet absorption spectrum were consistent with the results of the GC‐MS. Therefore, the experiment has shown that *A. polytricha* 5.584 could both degrade wheat bran fiber and effectively utilize the metabolites of wheat bran fiber.

### 3.7 Analysis of the effects of the fermentation products on rheological properties of dough sheet

**Figure 6:**
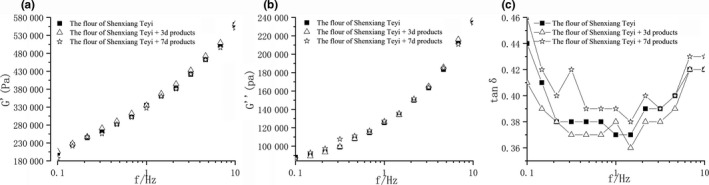
The frequency sweeping of dough sheet (a. the G' of dough shee; b. the G'' of dough sheet; c. the tanᵟ of dough sheet)

Rheological testing, particularly in the linear viscoelastic region, has been used to predict the structure and properties of dough and to research the functions of dough ingredients (Janssen et al., 1996; Miller & Hoseney, 1999)*.* The study simultaneously measures the elasticity (G′), viscosity (G″), and loss moduli (tan ᵟ) of three dough samples adding no fermentation products, fermentation products for 3 days, and fermentation products for 7 days, respectively (Figure 6).

During the process of dough‐mixing, gluten proteins are linked together via hydrogen bonds, disulfide bonds, and hydrophobic interactions to form strong cross‐links within and between polypeptide chains (Popineau et al., 1994; Wang et al., 2015). Overall, the results showed that G' and G'' increase gradually with the increase of frequency and the loss factor tanᵟ witnessed an initial decrease and then increase with the increase of frequency. After adding the fermentation products of wheat bran fiber by *A. polytricha* 5.584 to make dough sheet, there was no obvious difference among trends of G' and G'' of the three samples. However, the trends of changes in tanᵟ were apparent (Figure 6c). The tanᵟ of dough sheet decreased after adding the fermentation product for 3 days. Since the tanᵟ was G''/G', G'' increases less than proportionately compared to G'. On the contrary, the tanᵟ of dough sheet increased and was higher than that of the original dough after adding the fermentation product for 7 days, which indicated that G'' increases more than proportionately compared to G'. Previous research has shown that the tan ᵟ of dough was low for good quality flour (Song & Zheng, 2007). Therefore, on that basis, the quality of the dough sheet has been improved after adding the fermentation products of wheat bran fiber by *A. polytricha* 5.584 for 3 days. It is possible that the insertion of aromatic residue could improve the reactivity of amino groups on oligonucleotides when add the fermentation products (Kojima et al., 2006), resulting in the changes of hydrogen bonds, disulfide bonds, and hydrophobic interactions between gluten proteins.

## 4 CONCLUSION

In the study, the ability of *A. polytricha* 5.584 to degrade wheat bran fiber effectively was confirmed by *SEM*. The results of GC‐MS indicated that there were 9 kinds of aromatic compounds such as 3,5‐dimethylbenzaldehyde, 2‐methoxy‐4‐vinylphenol, 4‐hydroxy‐3‐methylacetophenone, 3,5‐dimethoxy‐4‐hydroxybenzaldehyde, diisooctyl phthalate, and 9‐octadecenamide being produced in the fermentation of wheat bran fiber. Among these products, diisooctyl phthalate and 9‐octadecenamide were proved to be metabolites. Furthermore, the results of the ultraviolet wavelength scanning indicated that the *A. polytricha* 5.584 could degrade vanillin, which further demonstrated that the strain has the ability to degrade wheat bran fiber. Presumably, macromolecular compounds in wheat bran fiber were decomposed into low molecular aromatic compounds through fermentation process by *A. polytricha* 5.584 and then further degraded or mineralized. Additionally, adding the products of wheat bran fiber fermented by *A. polytricha* 5.584 for 3 days could improve the elasticity of the dough sheet. As such, the expression results may help to lay the foundation for studying the degradation mechanism of wheat bran fiber by *A. polytricha*.

## CONFLICT OF INTEREST

We declare that we do not have any commercial or associative interest conflict for this work.

## ETHICAL STATEMENT

This study does not involve any human and animal testing.
